# Nutritional care competence among ICU nurses in China: a latent profile analysis

**DOI:** 10.1186/s12912-025-04244-w

**Published:** 2025-12-19

**Authors:** Xin-Yi Zhu, Xi-Xi Guo, Wen-Jie Ge, Zhi-Min Cao, Shou-Jun Zhu

**Affiliations:** 1 Department of Nursing & Department of Intensive Care Unit, The First Affiliated Hospital of USTC, Division of Life Sciences and Medicine, University of Science and Technology of China, Hefei, Anhui 230001 China; 2 College of Nursing, Bengbu Medical University, Bengbu, Anhui 233030 China; 3 Department of Nursing, The First Affiliated Hospital of USTC, Division of Life Sciences and Medicine, University of Science and Technology of China, Hefei, Anhui 230001 China

**Keywords:** Nurses, Intensive care unit, Nutritional care, Competence, Latent profile analysis

## Abstract

**Background:**

Nutritional care is essential in the treatment of critical patients, and the nutritional care competence among ICU nurses is a crucial skill in clinical practice of nutritional care for critically ill patients. Although previous studies have investigated the nutritional care competence of nursing staff, the investigation and heterogeneity analysis of nutritional care competence among ICU nurses in China are lacking.

**Aim:**

To investigate the current status of nutritional care competence among ICU nurses through latent profile analysis, identify potential subgroups and their population characteristics, and explore the factors that influence the potential subgroups.

**Methods:**

A cross-sectional and multi-center study of 561 ICU nurses in Anhui province was selected by convenience sampling method and surveyed with general information questionnaire and nutritional care competence scale for clinical nurses. Latent profile analysis (LPA) was used to identify potential subgroups among the nurses based on their competence in nutritional care. Logistic regression analysis was used to explore the factors associated with membership in different latent profiles.

**Results:**

The nutritional care competence among ICU nurses in Anhui Province was at an intermediate level and was categorized into three potential groups through latent profile analysis: low nutritional care competence group (31.73%), medium nutritional care competence group (48.84%), and high nutritional care competence group (19.43%). The results of logistic regression analyses showed that number of night shifts per month, job satisfaction, received regular nutritional care supervision, attended nutrition-related training, and received nutrition course education were the influencing factors of potential categories among ICU nurses’ nutritional care competence (*P* < 0.05).

**Conclusion:**

The nutritional care competence categorical characteristics among ICU nurses exhibit individual heterogeneity and could be categorized into three potential profiles. Nursing administrators should promptly identify and carry out targeted interventions according to the characteristics of nurses in different profiles to improve the overall quality of nutritional care.

## Background

Malnutrition is a prevalent and clinically significant issue among intensive care unit (ICU) patients, with an estimated incidence ranging from 38% to 78% [1]. This condition stems from the distinctive pathophysiology of acute critical illness, which is characterized by systemic inflammation, gastrointestinal dysfunction, and significant metabolic disturbances [2, 3]. These alterations collectively promote a state of hypercatabolism, leading to profound protein loss, muscle wasting, and weakness [4–6]. Malnutrition is independently associated with increased ICU length of stay (LOS), ICU readmission rate, incidence of infection (IOI), and risk of hospital mortality [7]. Therefore, nutritional risk assessment and individualized nutritional support strategies are essential for optimizing the prognosis of ICU patients.

Providing nutritional support delivers essential energy and nutrients, helps prevent vitamin and trace element deficiencies, and minimizes protein and muscle mass loss. This, in turn, mitigates the negative impacts of serious illnesses, such as infections, delayed healing, and higher mortality rates [8]. Adequate nutritional support can improve metabolic disorders in critically ill patients, stabilize the internal environment, maintain the structure and function of the intestinal mucosa, reduce the occurrence of complications, and shorten the length of hospitalization [9–11]. Providing nutritional care for critically ill patients in critical care necessitates a collaborative effort from a multidisciplinary team, including nutritionists, physicians, and nurses, to ensure that these patients receive consistent and effective nutritional management [12]. Although nutritional care may not be the primary focus of nurses, they play a crucial role in delivering clinical nutritional support. Their involvement significantly influences the effectiveness and quality of support through early nutritional risk screening, tailored nutritional interventions, ongoing evaluation of nutritional outcomes, complication prevention, and health education [13, 14]. Accurate and standardized nutritional care is essential to ensure the safety and effectiveness of nutritional therapy. Consequently, ICU nurses must possess advanced professional knowledge and specialized skills to deliver high-quality nutritional care for critically ill patients. Existing research indicates that ICU nurses frequently have insufficient knowledge and practical skills in nutrition care and that their practice is often guided by clinical experience rather than evidence-based approaches [15]. Consequently, enhancing the nutritional care competence of ICU nurses is crucial for improving the nutritional status of critically ill patients.

To help nurses identify areas of strength and weakness in nutritional care and improve nutritional care strategies, ZHU XY developed the Nutritional Care Competence Scale for Clinical Nurses (NCCS-CN) [16]. The NCCS-CN scale encompasses accurate nutritional risk screening, dietary assessment, and nutritional evaluation for patients, followed by the correct implementation of dietary guidance and nutritional support. The content is based on the Nutrition Care Practice Standards and Comprehensive Nurse Professional Performance Guidelines developed by the American Society for Parenteral and Enteral Nutrition [17]. Nutritional care competence refers to the comprehensive ability of nurses to perform accurate nutritional risk screening, dietary assessment, and nutritional evaluation, and to appropriately implement dietary guidance and nutritional support for patients, with the aim of effectively improving their nutritional status and promoting their health in clinical nursing practice [18]. With the introduction of the NCCS-CN scale, an increasing number of scholars have begun to focus on nurses’ nutritional care competence. Evidence suggests that such competence varies across clinical departments and that ICU nurses tend to have higher competence than nurses working in other areas, such as internal medicine [18]. Although existing studies have reported the competence level and influencing factors among general clinical nurses [16, 18], no comprehensive study has been undertaken to investigate this competence among ICU nurses. Furthermore, potential heterogeneous subgroups of nutritional care competence within the ICU nursing workforce have not received sufficient attention. To address this gap, a person-centered approach, such as latent profile analysis (LPA), could be beneficial.

The essence of LPA is a group classification technique that categorizes individuals based on their score differences in multidimensional indicators, aiming to achieve this through continuous data of observed variables, such as scale scores and behavioral indicators [19]. LPA can identify potential heterogeneous groups that are not directly observable in the samples, compensate for the limitations of traditional group classification, and help researchers analyze the characteristics and influencing factors of different groups [20, 21]. LPA has been widely applied in studies of nurses, including investigations into their psychological attributes, behaviors, and professional competencies [22–24]. LPA contributes to further research on the classifications, proportions, and characteristics of different potential profile populations, thus providing novel perspectives and methods for understanding and improving nurses’ competence more accurately. Previous studies have found that general demographic data, such as age, gender, and educational background, are influencing factors of nutritional care competence [18, 25], and job satisfaction is positively correlated with the professional ability of nurses [26]. Accordingly, all the above variables were considered for inclusion in the survey. This study used LPA to better understand the heterogeneity of ICU nurses’ nutritional care competence and the demographic differences related to this competence, which will help identify areas of strength and weakness in ICU nurses’ competence and develop improvement strategies.

This study aimed to identify heterogeneity groups of ICU nurses regarding nutritional care competence based on LPA and examine the sociodemographic correlations of these profiles. Therefore, this study adopted LPA to explore potential variations in nutritional care competence profiles, identify the traits of each profile, and compare personality traits across latent profiles. This study aimed to provide specific recommendations for interventions aimed at improving the nutritional care skills of ICU nurses, providing practical insights for policymakers and nurse practitioners.

## Methods

### Design

A cross-sectional descriptive study was conducted using a convenient cluster sampling method and adhered to the STROBE guideline for cross-sectional studies. Ethical approval for this study was approved by the Ethics Committee of the First Affiliated Hospital of the University of Science and Technology of China (Anhui Provincial Hospital) (2025-ky465).

### Participants

Nurses from 19 public general hospitals in Anhui Province who met the inclusion and exclusion criteria were selected to participate in a questionnaire survey using whole cluster sampling within each healthcare institution. Inclusion criteria: (1) ICU work ≥ 1 year; (2) hold a license to practice as a nurse; (3) informed consent and voluntary participation in this study. Exclusion criteria: (1) internship, advanced training, rotation nurses; (2) sick leave, vacation, or absentee nurses.

### Sample size

Based on the principle of Kendall’s method for sample size estimation, the sample size was calculated as 10 to 20 times the variable number [27]. A total of 22 variables were collected in this study(including 16 general information variables, 5 dimensions in the nutritional care competence scale for clinical nurses), taking into account the loss of 10% of the sample size. The formula for sample size calculation is *N* = 220÷(1–10%) ≈ 244. And the LPA requires a sample size greater than 250 [28], the sample size for this study was at least 500. Finally, a total of 561 ICU nurses were effectively investigated.

### Data collection

We distributed an online survey through the Questionnaire Star website. We explained the purpose of the survey, including the inclusion and exclusion criteria for survey respondents, to the head nurses of the ICU in each hospital over the phone and via WeChat before distributing the questionnaires. The head nurse of the hospital sent the QR code of Questionnaire Star through the WeChat platform to ICU nurses who met the criteria. All the participants responded to the online Questionnaire Star survey independently by scanning the QR Code via WeChat. The preamble of the questionnaire clarified the purpose, significance, target population, and method for completing the questionnaire. The investigation process followed the principles of informed consent, voluntariness, and anonymity. To avoid duplication, only one questionnaire could be completed per IP address. All items in the questionnaire were set as mandatory in Questionnaire Star, so participants must answer all questions before submitting the questionnaire. The questionnaires that takes less than 150 s to complete were considered invalid according to a cut score for response time at 2 s an item. The response times too slow were not been considered because many more factors may contribute to this situation. With a response rate of 97.79%, a total of 576 questionnaires were returned. There were no missing items in the 589 completed surveys because of the restriction of the answer system settings, but 28 of them were invalid and were removed from the dataset because of the detected all-the-same options. As a result, we received 561 valid questionnaires, and the effective response rate was 95.25%.

### Measures

#### General information questionnaire

The questionnaire was compiled by this group on its own clinical experience and literature review [18, 25, 26], it collected general information such as gender, age, marital status, years of work experience, title, education level, clinical teaching experience in the department, dietitian qualification, specialist nurse qualification, whether received regular nutritional care supervision, whether attended nutrition-related training, whether attended the training on nutritional care for critically ill patients, whether received nutrition course education, number of night shifts per month, job satisfaction, etc.

#### Nutritional care competence scale for clinical nurses

The Nutritional Care Competence Scale for Clinical Nurses (NCCS-CN) was developed in 2021 by the researcher [16]. Sixteen nursing professors or experts from six provinces in China were invited to participate in multiple rounds of expert correspondence. Using the convenience sampling method, two rounds of surveys were conducted among different clinical nurses who were tested for reliability and validity of the questionnaire, and the inclusion criteria for this study were clinical general nurses with a certificate of nursing practice. The purpose of the study was to develop a scale suitable for China’s national conditions to measure the nutrition care competence of clinical nurses. The scale comprises 5 dimensions: nutrition professional knowledge (7 entries), clinical nutrition assessment competence (10 entries), nutritional health education competence (13 entries), nutritional supportive care competence (18 entries), and nutritional care-related qualities (15 entries), totaling 63 entries. A 5-point Likert scale was used, ranging from 1 to 5, from “not at all compliant” to “fully compliant”, with a total score of 63 to 315. The higher the total score, the better the competence. The total Cronbach’s alpha coefficient of the scale was 0.991, the Cronbach’s alpha coefficients of the dimensions were 0.952–0.981, the retest reliability was 0.876, and the content validity index of the scale (S-CVI) was 0.968.

### Statistical analysis

Data were analyzed using SPSS 21.0 software, descriptive statistics for categorical variables in the general data were reported as frequency counts with percentages, the total scores followed an approximately normal distribution and were therefore expressed as mean ± standard deviation. Comparisons between groups were made using the Chi-square test or one-way ANOVA. Multicollinearity analysis was performed on statistically significant variables in the one-way analysis. AMOS 21.0 was used to conduct confirmatory factor analysis(CFA) to examine whether the construct of the instrument tested among Chinese ICU nurses would be in accordance with the original scale. Mplus 8.3 was used to analyze the latent profile model of ICU nurses’ nutritional care competence. The five-dimensional scores of the Nutritional Care Competence Self-Assessment Scale were used as exogenous indicators, and 1~5 profiles were selected sequentially for analysis. The three types of fitting indicators of the LPA were the Akaike Information Criterion (AIC), the Bayesian Information Criterion (BIC), and the adjusted BIC (aBIC). Lower values of AIC, BIC, and adjusted BIC (aBIC) indicate a better model fit. An Entropy value ranges between 0 and 1; closer to 1 indicates a more precise classification [29]. Corrected Likelihood Ratio (Lo-Mendell Rubin Likelihood Ratio Test, LMR) and Bootstrap Likelihood Ratio Test (BLRT) were used to compare the differences in model fit, and a statistically significant LMRT (*P* < 0.05) suggests that a model with K profiles is superior to a model with K-1 profiles [30]. After identifying the different categories of nutrition care competence among ICU nurses using LPA analysis, the variables with statistically significant differences among the subgroups were first analyzed using one-way analyses, such as the Chi-square test or one-way ANOVA (*P* < 0.05). Secondly, the variables with statistically significant differences mentioned above were included in the regression analysis, and logistic regression was used to analyze the influencing factors of various categories of nutritional care competence among ICU nurses, with a test level of α = 0.05.

## Results

### The validation results of the NCCS-CN among ICU nurses

In this study, the Cronbach’s alpha coefficient for the scale was 0.992, and the Cronbach’s α coefficient for the dimensions ranged from 0.958 to 0.986. The exceptionally high Cronbach’s α values (ranging from 0.952 to 0.991) raised concerns regarding potential item redundancy or insufficient discriminant validity among the subscales. To examine the latent factor structure and rigorously assess these psychometric properties, a confirmatory factor analysis (CFA) was performed. The model obtained a good fit. The χ^2^/df was 3.347, which is less than the strict index value of five (*P* < 0.001). The RMSEA was 0.065, which is less than 0.08. Other indices were: TLI = 0.918, CFI = 0.924, and NFI = 0.895. IFI = 0.924, RMR = 0.042.

### Results of latent profile analysis

The study conducted a profile analysis based on the NCCS-CN scores, and used AIC, BIC, aBIC, LMR (P-value), and BLRT (P-value) as the evaluation indices. When comparing models, if the AIC and BIC are smaller, the entropy is higher, and the LMR-p and BLRT-p values are less than 0.05, the model fit is better. As shown in Table 1, the number of model categories increased from 1 to 5, while the AIC, BIC, and aBIC continued to decrease, with entropy being the highest among the 4-profile models. The 4-profile solution was excluded due to a non-significant LMR test result (*p* > 0.05), along with no individual group exceeding 10% of the sample and substantial variation in total sample size across groups. Model 3 has ideal fit evaluation indexes and sample sizes for each group, and the probabilities of belonging to the three potential categories are 0.982, 0.981, and 0.982, respectively, indicating that the model is reliable for categorization, and the results are shown in Table 2. The minimum number of category samples in 3-profile is 109, accounting for 19.4% of the total samples, ensuring the stability of the results. Given that the 3-profile model aligns more closely with actual clinical practice, it was deemed the most preferable and optimal model. In conclusion, the 3-profile model is the best choice because of its excellent performance in terms of goodness-of-fit, classification significance, accuracy and stability.

**Table 1:** Potential profile analysis indicators (*n* = 561)

类别	AIC	BIC	ABIC	Entropy	LMR	BLRT	类别概率
1	20083.828	20127.125	20095.380	-	-	-	1
2	18631.386	18700.661	18649.869	0.916	<0.001	<0.001	0.374/0.626
3	17508.756	17604.010	17534.171	0.957	0.002	<0.001	0.488/0.317/0.194
4	17191.350	17312.582	17223.696	0.971	0.236	<0.001	0.023/0.307/0.483/0.187
5	16976.837	17124.048	17016.115	0.958	0.279	<0.001	0.025/0.175/0.160/0.453/0.187

**Table 2:** ICU nurses’ NCCS-CN potential profile category attribution probability matrix

Potential profile type	Probability of belonging to a potential category		
	C1	C2	C3
C1	0.982	0.011	0.008
C2	0.019	0.981	<0.001
C3	0.018	<0.001	0.982

Note. C1: Category 1 “Low nutritional care competence group”; C2: Category 2 “Medium nutritional care competence group”; C3: Category 3 “High nutritional care competence group”

**Fig. 1:**
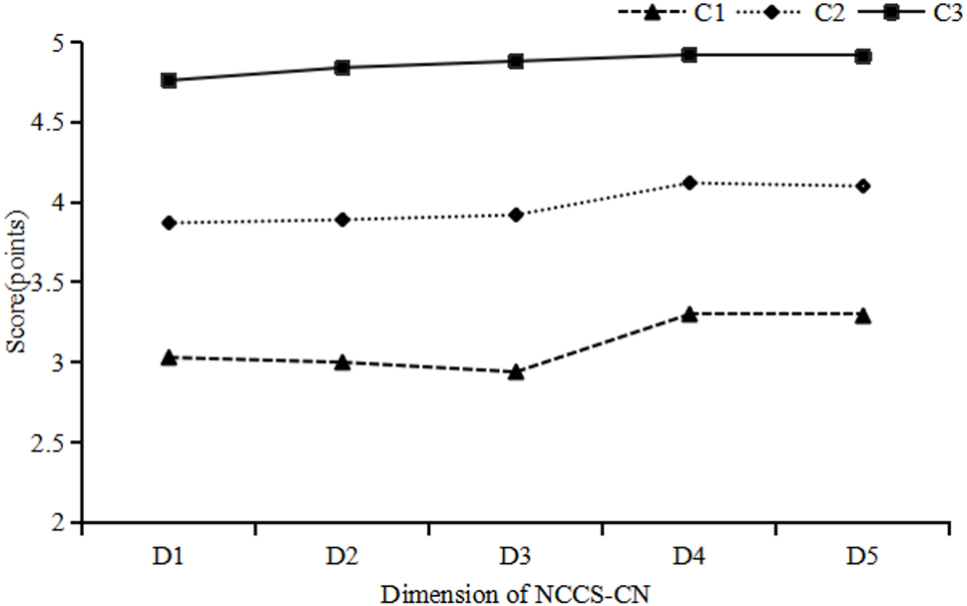
The distribution of the nutritional care competence among ICU nurses in the 3-profile model. C1: Category 1 “Low nutritional care competence group”; C2: Category 2 “Medium nutritional care competence group”; C3: Category 3 “High nutritional care competence group”. D1: Dimension 1 “nutrition professional knowledge”; D2: Dimension 2 “clinical nutrition assessment competence”; D3: Dimension 3 “nutritional health education competence”; D4: Dimension 4 “nutritional supportive care competence”; D5: Dimension 5 “nutritional care-related qualities”

**Fig. 2:**
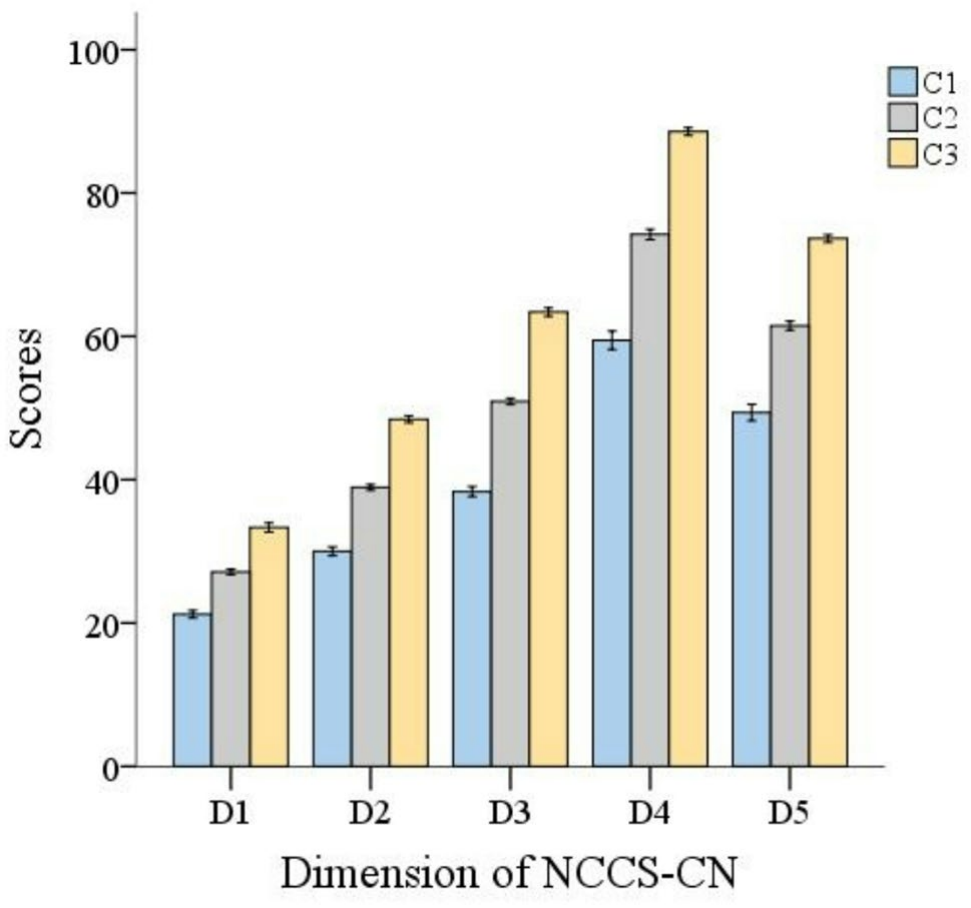
Mean scores on the dimensions of different nutritional care competence profiles

The total score for nutrition care competence among ICU nurses was 246.07 ± 41.48. The distribution of the nutritional care competence among ICU nurses in the 3-profile model is described in Fig. 1 and detailed in Table 3. Figure 2 illustrate the distribution probability of NCCS-CN among ICU nurses across various dimensions. Category 1 comprised 178 (31.73%) nurses who had the lowest scores in all dimensions, with a mean score of 198.36 ± 21.18, and was named the “Low nutritional care competence group”, represented by C1. Category 2 was the largest group, comprising 274 (48.84%) nurses with moderate scores on each dimension and was named the “Medium nutritional care competence group,” with a score of 252.68 ± 13.08, represented by C2. Category 3 formed the smallest group but demonstrated the highest level of nutritional care competence. It has 109 (19.43%) nurses and scored highly in all dimensions, being named the “High nutritional care competence group” with a score of 307.38 ± 9.87, which is represented by C3.

**Table 3:** Distribution of nutritional care competence in the 3-profle model (*N* = 561), mean (SD)

Variables	Total sample	C1(*n* = 178)	C2(*n* = 274)	C3(*n* = 109)
Total	3.91 ± 0.66	3.15 ± 0.34	4.01 ± 0.21	4.88 ± 0.16
Dimension 1	3.78 ± 0.77	3.03 ± 0.51	3.87 ± 0.45	4.76 ± 0.49
Dimension 2	3.79 ± 0.73	3.00 ± 0.40	3.89 ± 0.34	4.84 ± 0.25
Dimension 3	3.80 ± 0.74	2.94 ± 0.36	3.92 ± 0.28	4.88 ± 0.24
Dimension 4	4.02 ± 0.68	3.30 ± 0.48	4.12 ± 0.34	4.92 ± 0.16
Dimension 5	4.00 ± 0.69	3.29 ± 0.52	4.10 ± 0.35	4.91 ± 0.19

### Characteristics of latent profile membership

The results of univariate analysis showed that ICU nurses with different potential profiles were distributed differently in terms of education level, length of employment in ICU, number of night shifts per month, received regular nutritional care supervision, attended nutrition-related training, attended the training on nutritional care for critically ill patients, received nutrition course education, number of night shifts per month and job satisfaction, and the differences were statistically significant (*P* < 0.05), as detailed in Table 4. The main characteristics of the C3 were that it had the largest proportion of nurses with a master’s degree, consisted of fewer nurses employed in the ICU for less than ten years, received regular nutritional care supervision, had attended nutrition-related training, and had high job satisfaction. The resulting VIF values ranged from 1.04 to 1.27, affirming the absence of multicollinearity among these variables and thereby strengthening the overall robustness of the analytical model.

**Table 4:** Differences in latent traits of nutritional care competence among ICU nurses (*N* = 561), N (%)

Variables	Total sample(*n* = 561)	C1 (*n* = 178)	C2 (*n* = 274)	C3 (*n* = 109)	χ^2^	*P*
Gender					2.950	0.229
Male	127(22.64%)	48(26.97%)	58(21.17%)	21(19.27%)		
Female	434(77.36%)	130(73.03%)	216(78.83%)	88(80.73%)		
Age					8.631	0.195
< 30y	221(39.39%)	63(35.39%)	115(41.97%)	43(39.45%)		
30 ~ 39y	302(53.83%)	108(60.67%)	138(50.36%)	56(51.38%)		
40 ~ 49y	34(6.06%)	7(3.93%)	19(6.93%)	8(7.34%)		
≥ 50y	4(0.71%)	0(0.00%)	2(0.73%)	2(1.83%)		
Marital status					4.668	0.323
Unmarried	149(26.56%)	46(25.84%)	74(27.01%)	29(26.61%)		
Married	409(72.91%)	132(74.16%)	199(71.63%)	78(71.56%)		
Others	3(0.53%)	0(0.00%)	1(0.36%)	2(1.83%)		
Professional title					6.891	0.331
Registered nurse	78(13.90%)	17(9.55%)	43(15.69%)	18(16.51%)		
Senior nurse	232(41.35%)	81(45.51%)	105(38.32%)	46(42.20%)		
Charge nurse	234(41.71%)	76(42.70%)	115(41.97%)	43(39.45%)		
Associate professors of nursing or above	17(3.03%)	4(2.25%)	11(4.01%)	3(2.75%)		
Education level					34.558	< 0.001**
Junior college	62(11.05%)	18(10.11%)	30(10.95%)	14(12.84%)		
Undergraduate	481(85.74%)	157(88.20%)	242(88.32%)	82(75.23%)		
Master	18(3.21%)	3(1.69%)	2(0.73%)	13(11.93%)		
Length of employment in ICU					22.928	0.003**
<5y	188(33.51%)	59(33.15%)	90(32.85%)	39(35.78%)		
5 ~ 9y	179(31.91%)	66(37.08%)	92(33.58%)	21(19.27%)		
10 ~ 14y	151(26.92%)	44(24.72%)	68(24.82%)	39(35.78%)		
15 ~ 19y	32(5.70%)	8(4.49%)	20(7.30%)	4(3.67%)		
≥ 20y	11(1.96%)	1(0.56%)	4(1.46%)	6(5.50%)		
number of night shifts per month					13.704	0.033*
0	26(4.63%)	9(5.06%)	9(3.28%)	8(7.34%)		
1 ~ 5	159(28.34%)	48(26.97%)	91(33.21%)	20(18.35%)		
6 ~ 10	254(45.28%)	89(50.00%)	114(41.61%)	51(46.79%)		
>10	122(21.75%)	32(17.98)	60(21.90%)	30(27.52%)		
Received regular nutritional care supervision					31.367	< 0.001**
Yes	411(73.26%)	104(58.43%)	214(78.10%)	93(85.32%)		
No	150(26.74%)	74(41.57%)	60(21.90%)	16(14.68%)		
Attended nutrition-related training					19.498	< 0.001**
Yes	305(54.37%)	75(42.13%)	156(56.93%)	74(67.89%)		
No	256(45.63%)	103(57.87%)	118(43.07%)	35(32.11%)		
Attended training on nutritional care for critically ill patients					11.319	0.003**
Yes	504(89.84%)	149(83.71%)	256(93.43%)	99(90.83%)		
No	57(10.16%)	29(16.29%)	18(6.57%)	10(9.17%)		
Received nutrition course education at school					18.297	< 0.001**
Yes	395(70.41%)	107(60.11%)	197(71.90%)	91(83.49%)		
No	166(29.59%)	71(39.89%)	77(28.10%)	18(16.51%)		
Job satisfaction					68.914	< 0.001**
Dissatisfaction	34(6.06%)	20(11.24%)	11(4.01%)	3(2.75%)		
General satisfaction	314(55.97%)	120(67.42%)	162(59.12%)	32(29.36%)		
Satisfaction	213(37.97%)	38(21.35%)	101(36.86%)	74(67.89%)		

Note.∗means *p* < 0.05; ∗∗means *p* < 0.01

### Predictors of latent profile membership

We performed a multinomial logistic regression analysis to investigate whether different socio-demographic variables are associated with distinct profiles of nutritional care competence among ICU nurses. Variables that showed statistically significant differences in the univariate analysis were used as independent variables, while profiles were treated as dependent variables. The low nutritional care competence group was set as the reference group, and the reference category for each variable was the last set. The results are shown in Table 5. Nurses without night shifts were less likely to be in C2 (OR: 0.29, CI: 0.093 ~ 0.880, *p* < 0.05), and those with 1-to−5-night shifts per month were less likely to be in C3 (OR: 0.24, CI: 0.100 ~ 0.562, *p* < 0.05). Nurses who had attended nutrition-related training and received nutrition course education (OR: 2.17, CI: 1.164 ~ 4.039, *p* < 0.05, OR: 3.03, CI: 1.444 ~ 6.337, *p* < 0.01) were more likely to be classified into C3. Education level was a key factor, with nurses having junior college or undergraduate education background being less likely to be in the C3 (OR: 0.05, CI: 0.006 ~ 0.363, OR: 0.04, CI: 0.006 ~ 0.252, all *p* < 0.01). In comparison to the individuals in the C1, those who received regular nutritional care supervision and exhibiting job dissatisfaction or general satisfaction (all *P* < 0.05), were more likely to fall in both C2 and C3.

**Table 5:** Multinomial logistic regression analysis of potential categories of nutritional care competencies for ICU nurses (*n* = 561)

Variables	β	Waldχ^2^	*P*	OR	95%CI
**Profile 2 (vs. Profile 1)**					
Intercept	0.073	0.002	0.969		
Education level					
Junior college	0.472	0.123	0.726	1.60	0.115 ~ 22.445
Undergraduate	0.446	0.117	0.732	1.56	0.121 ~ 20.093
Master (refer)					
Number of night shifts per month					
0	-1.249	4.772	0.029*	0.29	0.093 ~ 0.880
1 ~ 5	-0.214	0.486	0.486	0.81	0.443 ~ 1.473
6 ~ 10	-0.439	2.471	0.116	0.65	0.373 ~ 1.114
>10 (refer)					
Job satisfaction					
Dissatisfaction	-1.156	6.518	0.011*	0.32	0.130 ~ 0.764
General satisfaction	-0.610	6.435	0.011*	0.54	0.339 ~ 0.871
Satisfaction (refer)					
Received regular nutritional care supervision					
Yes	0.601	6.547	0.011*	1.82	1.151 ~ 2.888
No (refer)					
Attended nutrition-related training					
Yes	0.302	1.887	0.170	1.35	0.879 ~ 2.080
No (refer)					
Received nutrition course education					
Yes	0.255	1.215	0.270	1.29	0.820 ~ 2.031
No (refer)					
**Profile 3 (vs. Profile 1)**					
Intercept	3.361	3.699	0.054		
Education level					
Junior college	-3.090	8.499	0.004**	0.05	0.006 ~ 0.363
Undergraduate	-3.266	11.504	0.001**	0.04	0.006 ~ 0.252
Master (refer)					
Number of night shifts per month					
0	-1.276	3.397	0.065	0.28	0.072 ~ 1.084
1 ~ 5	-1.438	10.704	0.001**	0.24	0.100 ~ 0.562
6 ~ 10	-0.615	2.877	0.090	0.54	0.265 ~ 1.100
>10 (refer)					
Job satisfaction					
Dissatisfaction	-2.359	10.648	0.001**	0.10	0.023 ~ 0.390
General satisfaction	-2.123	44.091	<0.001**	0.12	0.064 ~ 0.224
Satisfaction (refer)					
Received regular nutritional care supervision					
Yes	0.929	5.777	0.016*	2.53	1.187 ~ 5.402
No (refer)					
Attended nutrition-related training					
Yes	0.774	5.950	0.015*	2.17	1.164 ~ 4.039
No (refer)					
Received nutrition course education					
Yes	1.107	8.606	0.003**	3.03	1.444 ~ 6.337
No (refer)					

Note: *Refer* Reference category; *means *p* < 0.05; **means *p* < 0.01

## Discussion

Previous studies have typically relied on arbitrary cut-off scores or median splits on overall competence scales and have treated nurses as homogeneous groups. This study is the first to apply LPA to identify distinct profiles of nutritional care competence among ICU nurses. The results revealed clear hierarchical differentiation in competence and systematic variations between profiles regarding demographic characteristics and clinical experience. These differences provide empirical support for developing targeted interventions to enhance nutritional care competency. These findings offer novel insights that can inform tailored nutritional care education and training programs aimed at strengthening nutritional care competence based on the identified profiles.

### Potential characteristics of the NCCS-CN in ICU nurses and their application to nursing practice

The results revealed that the total score was 246.07 ± 41.48, and the overall average score was (3.91 ± 0.66), which is higher than that reported by Tang [18]. This discrepancy could be attributed to the different scopes of the studies: Tang’s study was conducted among nurses in various clinical departments, whereas our study focused on ICU nurses. Furthermore, our study results indicated that a higher proportion of nurses received nutrition-related training than those in Tang et al. ‘s study. This study identified three distinct groups: “Low nutritional care competence group” at 31.73%, “Medium nutritional care competence group” at 48.84%, and “High nutritional care competence group” at 19.43%. The finding that only 19.43% of nurses demonstrated an advanced competence level suggests a potential structural issue within the workforce and indicates a shortage of highly competent nurses. Nurses with advanced competencies are the core drivers of evidence-based practice, quality control, and technical innovation. Their low proportion contributes to the delayed recognition of nutrition-related complications and insufficient emergency responses, thereby increasing the nutritional care risks for critically ill patients [31, 32]. Schumacher [33] et al. advocated for competency-based medical education (CBME), a teaching approach that is patient-centered and learner-focused. This model centers on the delivery of high-quality care, which is achieved by tailoring instruction to individual needs and focusing on the attainment of specific competencies. This suggests that nursing managers should strengthen training for ICU nurses while also giving close attention to and addressing nurses’ needs. Moreover, nurses could constantly update their teaching and training methods and forms to enhance the internalization and absorption of their nutritional knowledge, thus improving their competence.

The “Low nutritional care competence group” accounted for 31.73% of the sample. These ICU nurses scored the lowest in all five dimensions of NCCS-CN. We found that the “nutritional health education competence” dimension was lower in C1 than in the other dimensions of the group. Nutritional health education competence is defined as the integration of knowledge, skills, attitudes, and values that nurses require to plan and implement effective educational programs for patients. This competence empowers patients to adopt the behavioral changes that promote positive health outcomes [34, 35]. Li et al. [34] found that the more years of nursing experience, the higher the health education competence of nurses, which is consistent with the characteristics of the C1 group nurses in our study. As depicted in Table 4, nurses in Group C1 were characterized by limited clinical experience in the ICU (less than 10 years). Makarem et al. [36] found that nurses with fewer years of experience tended to have limited experiential knowledge and skills. Consequently, they require additional support and training in health education to ensure adequate competence. Nurses with a strong foundation of nutritional health knowledge are better equipped to address patient needs, provide tailored nutritional health education interventions, promote nutritional health literacy, and improve nutritional health education competence [37]. Therefore, developing a training system that focuses on nutritional health knowledge and skills for nurses with low competence may be a key priority for nursing administrators.

The “Medium nutritional care competence group” comprised 48.84% of the participants, and the nurses in this group did not exhibit significant characteristics in their general demographic profile. Notably, the score for the nutrition professional knowledge dimension was the lowest, indicating that nutrition professional knowledge was a weakness for nearly half of the ICU nurses. Similarly, current study highlighted that nutrition knowledge was a weakness of ICU nurses [38]. Most nurses in C2 attended training on nutritional care for critically ill patients and received nutrition education at school. Our analysis of the data for this dimension indicated that the nurses in this group exhibited a less comprehensive understanding of essential nutrition knowledge. This knowledge, typically acquired through formal coursework, encompasses the principles of nutrition, dietary guidelines for the public, and disease-specific nutritional information. Recent research has revealed that the nutrition education provided in undergraduate nursing programs is inadequate for preparing nurses to deliver nutrition care that meets the needs of their patients or themselves as future healthcare professionals [39]. Nurses’ knowledge levels can be shaped by their interest in nutrition educational programs and experience, and a need for customized education and training, even among experienced nurses [38]. Therefore, it is crucial to provide tailored educational approaches for ICU nurses, such as “Internet,” “science courses,” “nursing education or schools,” and “communication with peers” [40].

Nurses in the “High nutritional care competence group” exhibited the highest level of competence, accounting for 19.43% of the total respondents. Nurses in this group scored the highest in all dimensions of the NCCS-CN. Notably, nurse managers scored the lowest in the dimension of nutritional professional knowledge and the highest in the dimension of nutritional support care competence. Although nurses with advanced skills may have developed effective “practical wisdom” through years of clinical experience, they might struggle to articulate the reasons behind their actions using their professional expertise, and their operational processes and decisions may be based on empirical cognitive patterns rather than entirely derived from the memory of book theories. However, adequate clinical nutritional knowledge is an indispensable component of clinical nutritional practice [41]. The International Council of Nurses emphasized that advanced nursing professionals should possess a wide-ranging theoretical foundation and substantial practical experience and play crucial roles in evidence-based nursing practice [42]. Furthermore, decisions regarding nutritional therapy should adhere to the principles of evidence-based medicine, which is not possible without updating knowledge reserves [43]. Given that nurses in C3 show high overall competence yet gaps in specialized knowledge, we recommend that nurse managers implement evidence-based educational interventions (including virtual courses, in-person training, and webinars) will enhance nurses’ nutritional knowledge.

### Associations among NCCS-CN profiles and associated predictors

Our study also revealed that the three classes differed in terms of their demographic and other characteristics. According to the multinomial logistic regression analysis, demographic characteristics did not significantly impact the nutritional care competence of ICU nurses, except for their education level. The results indicated that nurses with a master’s degree were more likely to be classified into the C3 group. This could be attributed to the fact that the Master’s program in nursing enhanced their comprehensive competencies, enabling them to provide more qualified and evidence-based nursing for the benefit of clinical practice [44]. Comparing groups C2 and C3 with group C1, it was found that the frequency of night shifts per month, levels of job satisfaction, regular oversight in nutritional care, participation in nutrition-related training sessions, and education in nutrition courses were all significant factors. Nurses who worked more night shifts were more likely to be classified into the C3 group. This observed association may be attributable to the high work pressure of ICU night shifts, which frequently involve complex emergencies and sudden complications, thereby facilitating the accumulation of substantial nursing experience and competence [45]. However, night shifts are a key factor that may lead to nurses’ emotional exhaustion [46], and the frequency of night shifts, short recovery periods, and irregular rotation patterns impact the distinct dimensions of burnout [47]. Moderate night shifts may be associated with improved competence. Nursing managers should set a scientific upper limit for the number of night shifts based on actual circumstances, optimize the shift scheduling model, and strengthen the guarantee of night shift resources to prevent the enhancement of competence from turning into occupational burnout and decline in competence due to excessive fatigue. The results indicated that nurses with low job satisfaction exhibited a relatively weak level of nutritional care competence. Prior studies have demonstrated that improvements in work ability levels and increases in Job satisfaction and task performance are similarly correlated [48, 49]. A study by Wang et al. [50], focusing on newly recruited nurses, demonstrated that job satisfaction serves as a significant motivator for work engagement and constitutes a critical determinant of core nursing competence. Job satisfaction is defined as an individual’s capacity to fulfill their needs, goals, values, and beliefs. When ICU nurses face limitations in their clinical practice, their job satisfaction tends to decrease accordingly [51]. Hospitals should create a supportive work environment, provide opportunities for relaxation, and improve overall well-being, leveraging the role of social support to reduce emotional exhaustion and enhance nurses’ job satisfaction [52]. Furthermore, it is important to highlight that most nurses in C3 regularly received guidance on nutritional care and engaged in training related to nutrition. During their school years, they also received nutritional education. Clinical supervision focuses on problem resolution, which can help nurses perform their work more effectively, thereby addressing the increasing number of nursing issues [53]. Moreover, a comprehensive nutritional education curriculum can equip medical students with the skills necessary to engage in high-quality nutritional practices. Accordingly, integrating comprehensive nutrition education into medical training is imperative [54, 55].

### Relevance to clinical practice

This study provides insights for nursing managers and policymakers to develop training programs that enhance ICU nurses’ competence. The findings of this study indicate that ICU nurses’ nutritional care competence is closely associated with several factors: receipt of regular nutritional care supervision, participation in nutrition-related training, prior nutrition coursework, educational level, monthly number of night shifts, and overall job satisfaction. Hence, nurse managers must consider these subgroup characteristics when tailoring advanced practice programs for ICU nursing. First, nutrition training programs should be established in clinical hospitals to extend the nutrition education initiated during undergraduate studies and meet nutrition education requirements across the entire continuum of medical training [56]. Nurse managers could develop nutritional care training programs that cater to nurses with varying education levels and implement a structured peer teaching program, whereby nurses with high competence demonstrate and supervise to improve the operational skills and knowledge of low-competence nurses [57]. Additionally, a regular nutrition care supervision system should be established within the departments. Implementing structured clinical supervision in nursing helps improve the quality of nutritional care and promotes the ongoing standardization of nursing practices [58]. Nurse managers should adopt regular nutritional care supervision strategies and implement supervision processes to overcome obstacles in nutritional care and improve nutritional care practice. Third, nursing managers should optimize human resource allocation and the organization of night shifts. They should critically examine the relationship between night shift work and nurses’ competence and develop shift schedules that consider both competence levels and fatigue. Moreover, hospitals should promote a positive work environment to strengthen nurses’ commitment and their job satisfaction. Nursing managers and policymakers are encouraged to establish incentive systems that recognize exceptional performance in specialized areas such as nutritional care, for example, through financial incentives or opportunities for career advancement [23]. By implementing these targeted interventions, healthcare organizations can unlock the nutritional care potential of clinical nurses and cultivate a work culture that actively encourages nutritional care.

### Strengths and limitations

This study has the following strengths. This study is the first to employ LPA to clarify the heterogeneity in the levels of nutritional care competence and its specific distribution among nurses. Second, this study was based on a cross-sectional and multicenter study of 561 ICU nurses, which enhanced the reliability and representativeness of the findings. This study has several limitations. First, this competence was assessed using a self-reported online questionnaire, which is inherently subject to participants’ subjective perceptions and may be influenced by individual cognitive biases, thereby affecting the accuracy and authenticity of the data [23]. Future studies should incorporate objective assessment tools to link latent profile membership with observed performance indicators and employ mixed-methods designs to explore the underlying determinants of different competence patterns. Second, the use of a convenience sampling strategy within a single Chinese province limits the representativeness of the sample at the national level and may have introduced a selection bias [59]. Future research should validate these findings across diverse geographic and institutional settings. Third, additional variables may have influenced this competence of ICU nurses. However, the present study considered only sociodemographic and job-related characteristics and did not examine underlying theoretical mechanisms, such as self-efficacy and organizational support, or specific clinical outcomes associated with the identified profiles of nurses. Future research could be guided by relevant theoretical frameworks to explore the relationships between this competence and psychosocial and organizational factors. Fourth, the cross-sectional design precludes inferences about the causal relationships among the variables, and the current findings should primarily be viewed as hypothesis-generating. Longitudinal studies that track changes in nurses’ nutritional care competence over time are particularly valuable for clarifying causal pathways. Lastly, because the first year of nursing practice in China typically involves rotational training, nurses in their first year were excluded. This strengthened the focus on a stable ICU workforce but limited the generalizability of the findings to novice nurses, a population that warrants targeted investigation in future studies.

## Conclusion

This study employed LPA to identify distinct patterns of nutritional care competence among ICU nurses and examine the associated influencing factors. Nutritional care competence was best described by a three-profile model comprising a “Low nutritional care competence” group (C1, 31.73%), a “Medium nutritional care competence” group (C2, 48.84%), and a “High nutritional care competence” group (C3, 19.43%). Nurses in C3 demonstrated the highest scores across all dimensions of this competence, while those in C1 exhibited the lowest scores. The factors associated with profile membership included education level, number of night shifts per month, job satisfaction, receipt of regular nutritional care supervision, participation in nutrition-related training, and completion of a nutrition course. It is recommended that nursing managers should recognize these differences in competence and implement targeted, profile-based management and educational strategies to enhance ICU nurses’ nutritional care competence, thereby ensuring the quality of nutritional care. Future research could explore the mechanisms underpinning high levels of competence and identify additional factors that support or hinder the development of such proficiency. The present findings provide an empirical foundation for designing and implementing tailored education and training programs aimed at strengthening nutritional care competence among ICU nurses.

## Data Availability

All data generated or analysed during this study are included in this published article and its supplementary information files.
